# Testicular sperm retrieval for intracytoplasmic sperm injection: when to consider it after unsuccessful intracytoplasmic sperm injection with ejaculated sperm?

**DOI:** 10.1111/andr.13643

**Published:** 2024-04-02

**Authors:** Muhammed Arif Ibis, Eda Ureyen Ozdemir, Khaled Obaid, Cagri Akpinar, Batuhan Ozmen, Kaan Aydos, Onder Yaman

**Affiliations:** ^1^ Department of Urology Ankara University School of Medicine Ankara Turkey; ^2^ Center for Research on Human Reproduction Ankara University School of Medicine Ankara Turkey; ^3^ Department of Obstetrics and Gynecology Ankara University School of Medicine Ankara Turkey

**Keywords:** ejaculated spermatozoa, ICSI, male infertility, sperm DNA fragmentation, sperm retrieval, testicular spermatozoa

## Abstract

**Background:**

The question of whether patients are more likely to succeed with testicular sperm intracytoplasmic sperm injection (T‐ICSI) after unsuccessful ICSI with ejaculated sperm (Ej‐ICSI) remains unknown.

**Objective:**

The study aimed to identify potential predictors of successful T‐ICSI in men with idiopathic infertility and oligozoospermia (sperm concentration < 15 × 10^6^/mL, non‐azoospermic) who had previously experienced unsuccessful Ej‐ICSI.

**Materials and methods:**

In total, 154 couples with male partners who had oligozoospermic conditions after two unsuccessful cycles of Ej‐ICSI switched to T‐ICSI. Before initiating T‐ICSI, the sperm DNA fragmentation index (DFI) was assessed in ejaculated specimens. Participants were divided into two groups: group A (live birth (+), *n *= 60) and group B (live birth (−), *n* = 94).

**Results:**

Fertilization, clinical pregnancy, live births, and miscarriages had rates of 72.7%, 44.2%, 39%, and 5.2%, respectively. The total motile sperm (TMS) count in group A was significantly higher (3.8 ± 1.5 million) than in group B (3 ± 1.6 million; *p* = 0.002). DFI was significantly higher in group A (24.2 ± 12.3) than in group B (18.1 ± 11; *p *= 0.001). Hormone levels and oocyte counts showed no statistically significant differences between groups. Multivariate regression analysis revealed that TMS (odds ratio [OR]: 1.46; 95% CI, 1.14–1.87, *p* = 0.003) and DFI (OR: 1.04; 95% CI, 1.01–1.08, *p* = 0.009) were found to be significant predictors of live birth outcomes. At a cutoff point of 2.55 (area under the curve [AUC] = 0.65), the optimal sensitivity and specificity values for TMS were 78% and 48%, respectively. At a cutoff point of 25.8 (AUC = 0.65), DFI had a maximum sensitivity of 51.7% and a specificity of 78.7%.

**Conclusions:**

TMS and DFI were found to be significant predictors of live birth outcomes in couples with oligozoospermic male partners undergoing T‐ICSI. These findings may help clinicians tailor treatment strategies for this specific patient population.

## 1 INTRODUCTION

Since its introduction in 1992, intracytoplasmic sperm injection (ICSI) has been a significant invention in achieving biological paternity for men with azoospermia, particularly when combined with surgical testicular sperm retrieval.^1^ Currently, using testicular sperm when no sperm is present in the ejaculate remains a necessary and acceptable practice.

However, there is no consensus among experts on the necessity of using testicular sperm for ICSI cycles in men with oligozoospermia.^2^ Because the available data are limited, the supporting evidence for the beneficial use of testicular spermatozoa in ICSI procedures involving non‐azoospermic individuals is limited to particular scenarios, primarily cases where excessive sperm DNA damage has been unambiguously confirmed in ejaculated spermatozoa.^3^

Several studies have shown that sperm DNA integrity plays a role in the etiology of male factor infertility.^4^ It has recently been suggested that the sperm DNA fragmentation rate is more indicative of male reproductive success than routine semen analysis.^5^ The sperm DNA fragmentation index (DFI) significantly impacts the clinical course of in vitro fertilisation (IVF). Low blastocyst development rates after ICSI are attributed to high DFI.^6^ Meanwhile, the results suggested that ejaculate sperm may be more susceptible to DNA damage caused by oxidative stress during epididymal passage.^7^ During their epididymal passage following testicular maturation, sperm cells undergo some epigenetic modifications and protein composition rearrangements.^8^ This process is required for DNA stability and the development of oxidative stress resistance, and changes in the epigenetic profile of mature sperm are closely associated with male factor infertility.^9^, ^10^ This has led to the hypothesis that sperm DNA damage could be a contributing factor to the failure of ICSI cycles with ejaculated sperm (Ej‐ICSI). Although studies on this hypothesis have been conducted, the heterogeneity and limitations of the studies have resulted in the need for further studies to support the hypothesis.^7^, ^11^, ^12^

This study aimed to explore the potential predictors of successful ICSI cycles using testicular sperm (T‐ICSI) in men with oligozoospermia who had previously experienced unsuccessful Ej‐ICSI cycles.

## 2 MATERIALS AND METHODS

We conducted a retrospective analysis using a database of 154 couples with ICSI treatment between January 2019 and August 2022. The study was approved by the Ethics Committee of the Faculty of Medicine, Ankara University (registration number: i07‐500‐23).

The inclusion criteria of this study include (a) infertile couples who had two previous unsuccessful Ej‐ICSI cycles, (b) couples who used testicular sperm in the subsequent ICSI cycle, (c) men with idiopathic oligozoospermia (< 15 × 10^6^/mL), (d) patients with DFI measured after unsuccessful second ICSI cycles, and (e) patients who adhered to the follow‐up schedule. The exclusion criteria include (a) men with azoospermia and normozoospermia; (b) men with any other cause of infertility abnormalities in their medical history, such as abnormal physical examination (clinical varicocoele or solitary testicle), endocrine profiles (hypogonadotropic hypogonadism, or hyperprolactinemia), or genetic disease (Kallmann syndrome or Klinefelter syndrome); (c) couples with medically proven female partner infertility factors, including advanced female age over 35 years, apparent infertility factors, such as closed fallopian tubes, uterine abnormalities, polycystic ovary syndrome, adenomyosis, endometriosis, hydrosalpinx, and female principal follicle‐stimulating hormone (FSH) level over 10 IU/L, and anti‐Müllerian hormone (AMH) less than 1.2 ng/mL.

All patients with spermatozoa count less than 10 and 5 million/mL underwent karyotype analysis and Y‐chromosome microdeletion testing. After the second unsuccessful Ej‐ICSI cycle, DFI was measured from the ejaculated specimen. Men with DFI > 20.0% were treated with antioxidants for at least 3 months. The oral antioxidant treatment included a combination of commercially available vitamins (C and E) and supplements (folic acid, selenium, and zinc). The DFI measurement of these patients was re‐evaluated after at least 3 months of treatment, during which they continued to receive the oral antioxidant therapy. Testicular sperm aspiration (TESA) for the T‐ICSI cycle was performed on the same day as oocyte retrieval or 1 day before oocyte retrieval, depending on the preference of our embryologist.

### 2.1 Semen analysis

Semen samples were obtained from the volunteers on‐site at the clinic through masturbation into a sterile container, following a recommended period of sexual abstinence of between 3 and 5 days. In summary, after a 30‐min liquefaction period (for raw semen) or incubation at 37°C for 5 min (for liquefied samples), the sperm volume was measured using a 10 mL sterile serological pipette. A 5 µL semen drop was placed onto the application area of a Makler counting chamber (Sefi Medical Instruments Ltd., Haifa, Israel) following gentle pipetting of the sample. A 0.1 mm^2^ smear was obtained to assess sperm concentration and motility. The concentration of the sample was determined by counting sperm heads in successive 10 squares, with the mean calculated after counting five different 10‐square‐in‐rows. Motility was assessed using the same chamber, counting a minimum of 200 spermatozoa per sample and calculating the sum of the rapid progressive sperm percentage. All evaluations were conducted under a phase‐contrast microscope (Nikon, Germany) at 200× magnification. Sperm morphology was examined after creating 10 µL sperm smears on glass slides, fixing them in 100% methanol for 10 min. Diff‐Quik stain (RAL Diagnostic, Martillac, France) was then applied, and sperm morphology was evaluated under a 100× objective, classifying 200 sperm as either having abnormal or normal morphology according to the Tygerberg strict criteria.

### 2.2 DNA fragmentation analysis

The DFI was determined in this study using the terminal deoxynucleotidyl transferase dUTP nick‐end labeling (TUNEL) assay. The methodology included several steps, starting with sample fixation and then permeabilization. After three washes in phosphate‐buffered saline, the samples were fixed in a 4% paraformaldehyde solution and embedded in paraffin before sectioning. The tissue sections were then deparaffinized and rehydrated. The tissue sections were treated with a TUNEL reaction mixture containing TdT enzyme and fluorescein‐labeled dUTP to label the DNA breaks. During the incubation period, resuspension was accomplished using a permeabilization solution containing Triton X. The labeling was performed using the manufacturer's specific “TUNEL reaction mixture.” In each reaction set, negative and positive samples were prepared using an agent that caused severe DNA fragmentation for the internal control procedure. TUNEL‐positive cells then emitted green fluorescence, which was detected using a fluorescence microscope. The DFI was then calculated by determining the ratio of TUNEL‐positive cells to the total number of cells, yielding a quantitative measure of DNA fragmentation in the samples. To achieve accurate and reliable results, a minimum of 500 cells were counted for each sample.

### 2.3 TESA procedure

TESA was performed, as reported previously.^13^ It began with the administration of local anesthesia (2% prilocaine, 5 mL) near the spermatic cord. The center, upper, and lower poles of each testicle were then aspirated using a 23‐G needle attached to a 10‐mL syringe. Throughout the procedure, a constant negative pressure was maintained in the syringe as the needle reached the testis. Applications were done unilaterally in all patients. Aspirate samples were examined by the embryologist under an inversion microscope in the operating room. The TESA procedure's success depended on obtaining an adequate quantity of spermatozoa with normal morphology. Once this criterion was met, the TESA procedure was considered successful and concluded. The TESA procedures were regularly performed by a single experienced urologist. All patients provided the informed consent.

### 2.4 Ovarian stimulation and oocyte collection

All patients had basal transvaginal ultrasonography and serum FSH and estradiol (E2) level assessments on their menstrual cycles’ second or third day. Controlled ovarian hyperstimulation with recombinant FSH at a dose of 150−225 IU/day was initiated for the patients based on their age, body mass index, antral follicle count, and AMH values. Serial transvaginal ultrasonography measurements and serum E2 levels were used to monitor follicular growth, and the dosage was adjusted individually based on ovarian response.

A 0.25 mg/day gonadotropin‐releasing hormone antagonist was administered and continued throughout the ovarian stimulation when a follicle reached an average diameter of 14 mm on ultrasound and/or serum luteinizing hormone (LH) levels reached > 10 IU/L. Human chorionic gonadotropin (hCG; Ovitrelle 250 µg, Merck Serono, Modugno, Italy) was administered when serum E2 levels reached ≥500 pg/mL and at least three follicles measured ≥17 mm. The oocyte retrieval procedure was performed under transvaginal ultrasound guidance approximately 34−36 h after the hCG administration. The ICSI procedure was used for fertilization.

Embryos were categorized according to their morphological appearances (size and shape of blastomeres, degree of fragmentation, multinucleation, and cytoplasm appearance): grade 1 (highest) to grade 4 (lowest), and excellent quality (grades 1−2) embryos were selected for transfer. Embryo transfer was conducted under ultrasound guidance on the third day after oocyte retrieval. Due to national regulations on embryo transfer, only one embryo was used in the first two procedures, whereas two embryos were used in the third procedure.

All patients received luteal phase support with vaginal progesterone 400 mg twice a day from the day of oocyte retrieval to the pregnancy test 12 days after embryo transfer. Support was continued until a negative pregnancy test result was obtained or until the 12th week of pregnancy. Patients with a β‐hCG level > 10 IU/mL were considered pregnant.

Clinical pregnancy was defined as the presence of serum β‐hCG levels 2 weeks after embryo transfer and the presence of fetal heartbeat at the 6th week of pregnancy. Miscarriage was defined as the loss of an embryo or fetus, as well as all or part of its appendages, before the 20th week of pregnancy or weighing less than 500 g. Live birth was defined as the delivery of a live infant after the 24th week of pregnancy. For each patient, the implantation rate was calculated by dividing the number of gestational sacs by the number of transplanted embryos and multiplying by 100.

### 2.5 Statistical analysis

Descriptive statistics have been presented as percentages for categorical variables, whereas continuous variables have been summarized using measures such as mean, standard deviations, median, and the 25th and 75th percentiles. For continuous variables with a normal distribution, independent *t* tests were used to compare differences between groups in demographic and clinical characteristics. For continuous variables that were not normally distributed, the Mann–Whitney *U*‐test was used. The chi‐square test was used to compare categorical variables. Multivariate logistic regression analysis was conducted to evaluate the associations between various factors (variables with *p* < 0.250 difference in univariate analysis between two groups) and the likelihood of achieving a successful live birth. Odd ratios (ORs) and 95% confidence intervals (95% CIs) were calculated to quantify the strength of these associations. The predictive accuracy of total motile sperm (TMS) and DFI for live birth outcomes was evaluated using a receiver operating characteristic (ROC) curve analysis. The optimal sensitivity and specificity values were obtained by identifying the cutoff points that maximized sensitivity and specificity. The area under the curve (AUC) was calculated to evaluate the overall performance of the predictive models.

## 3 RESULTS

This study included 154 couples. The average age of female and male participants was 30.1 ± 4.2 and 33.7 ± 3.8 years, respectively. The mean female FSH level was 6.7 ± 1.4 IU/L, and the average AMH level was 2.4 ± 0.9 ng/mL.

The results of the semen analysis showed a concentration of 7.9 ± 3.7 million sperm/mL and a TMS of 3.3 ± 1.6 million motile sperm per ejaculate. The mean sperm morphology was 2.8 ± 1.1, and the DFI was 20.5 ± 11.9. The mean total oocytes retrieved was 9.9 ± 2.9, whereas the mean mature oocytes was 8.1 ± 2.2. Fertilization was successful in 72.7% of cases, whereas implantation was achieved in 49.4% of the total attempts. Clinical pregnancies were observed in 44.2% of cases, resulting in 68 successful pregnancies and 60 live births. Miscarriages were reported in eight cases, accounting for 5.2%.

Participants in this study were divided into two groups: group A (live birth (+)) and group B (live birth (−)). TMS count in group A was significantly higher (3.8 ± 1.5 million) than in group B (3 ± 1.6 million; *p* = 0.002). Additionally, group A had a higher sperm morphology (2.9 ± 0.9) than group B (2.7 ± 1.2); however, the difference was not statistically significant (*p* = 0.221). Moreover, group A had significantly higher DFI (24.2 ± 12.3) than group B (18.1 ± 11; *p* = 0.001). In terms of male hormone levels, there was no statistically significant difference between groups A and B in FSH, LH, and total testosterone levels (*p* = 0.442, *p* = 0.292, and *p* = 0.811, respectively). Female age was comparable between groups (*p* = 0.118). Furthermore, when it comes to female hormone levels, there were no statistically significant differences in FSH and AMH levels between groups A and B (*p* = 0.271 and *p* = 0.378, respectively). The total number of oocytes retrieved in group A was 10.3 ± 2.9, whereas group B yielded 9.6 ± 2.8 oocytes (*p* = 0.112). In terms of mature oocytes, group A had 8.2 ± 2, whereas group B had 8 ± 2.4 (*p* = 0.663). Comparisons between groups are summarized in Table 1.

**TABLE 1:** Demographic and clinical characteristics and reproductive outcomess stratified by live birth.

	Group A (*n* = 60) Mean ± SD Median (25%–75%)	Group B (*n* = 94) Mean ± SD Median (25%–75%)	*p*‐value
*Female*			
Age (years)	29.4 ± 3.5 30 (27−32)	30.5 ± 4.5 31 (28−34)	0.118
BMI (kg/m^2^)	25.3 ± 3.2 25.8 (23.7−27.4)	26.2 ± 4.7 25.3 (23.6−29.5)	0.160
Smoking, *n* (%)	9 (15)	13 (13.8)	0.840
FSH (IU/L)	6.8 ± 1.5 7.1 (5.6−8.1)	6.6 ± 1.4 7.0 (5.4−7.9)	0.271
AMH (ng/mL)	2.3 ± 0.8 2.5 (1.6−3)	2.5 ± 0.9 2.6 (1.8−3.1)	0.378
AFC	9.2 ± 2.8 9 (7−11)	8.7 ± 3.1 8 (6−10)	0.304
Total oocytes, *n*	10.3 ± 2.9 10 (8−12)	9.6 ± 2.8 9 (7−11)	0.112
Mature oocytes, *n*	8.2 ± 2.0 8 (7−10)	8.0 ± 2.4 8 (6−10)	0.663
2PNs, *n*	6.6 ± 2.8 7 (4−9)	6.1 ± 2.3 6 (4−8)	0.216
Endometrial thickness (mm)	8.4 ± 1.3 8.3 (7.3−9.2)	8.7 ± 1.3 8.6 (7.6−9.5)	0.197
*Male*			
Age (years)	33.2 ± 4.1 33 (30−36)	34.0 ± 3.7 34 (31−36)	0.208
BMI (kg/m^2^)	28.8 ± 5.2 29.2 (25.8−32.3)	30.0 ± 6.0 30.8 (25.7−34.4)	0.207
Smoking, *n* (%)	19 (31.7)	22 (23.4)	0.258
Testicular volume (mL)	19.2 ± 3.0 19 (17−21)	18.9 ± 2.9 19 (17−21)	0.468
Subclinic varicocoele, *n* (%)	4 (6.7)	7 (7.4)	0.855
Semen volume (mL)	3.1 ± 1.0 3.1 (2.4−3.8)	3.0 ± 1.0 2.9 (2.4−3.6)	0.561
Concentration (sperm/mL)	8.5 ± 4.1 8.4 (5.6−12.1)	7.5 ± 3.5 7.4 (5.1−10.2)	0.115
TMS, million motile/ejaculate	3.8 ± 1.5 3.7 (2.7−4.9)	3.0 ± 1.6 2.9 (1.9−4.2)	0.002
Morphology (%)	2.9 ± 0.9 3 (2−3)	2.7 ± 1.2 3 (2−3)	0.221
DFI	24.2 ± 12.3 26.1 (15.7−30.9)	18.1 ± 11.0 17.1 (10.2−24.9)	0.001
FSH (IU/L)	6.2 ± 3.0 6.0 (4.4−8.1)	5.8 ± 3.2 5.6 (3.4−8.2)	0.442
LH (IU/L)	5.5 ± 1.7 5.5 (4.2−6.4)	5.9 ± 2.8 5.7 (4.0−7.6)	0.292
Total testosterone (ng/dL)	401.3 ± 108.9 410.5 (337‐ 481)	397.5 ± 87.8 396.0 (337.6−463)	0.811
Antioxidant use, *n* (%)	34 (56.7)	45 (47.9)	0.287
*Reproductive outcomes*			
Fertilization (%)	74.1	71.8	0.266
Implantation, *n* (%)	60 (100)	16 (17)	0.001
Positivity of ß‐hCG, *n* (%)	60 (100)	16 (17)	0.001
Clinical pregnancy, *n* (%)	60 (100)	8 (8.5)	0.023
Live birth, *n* (%)	60 (100)	0 (0)	<0.001
Miscarriages, *n* (%)	0 (0)	8 (8.5)	0.023

Abbreviations: BMI, Body mass index; FSH, follicle stimulating hormone; AMH, anti‐mullerian hormone; AFC, antral follicle count; PNs, pronuclei; TMS, total motile sperm; DFI, DNA fragmentation index; LH, luteinizing hormone;  β‐hCG: Beta‐human chorionic gonadotropin.

A multivariate regression analysis investigated the potential associations between various factors and the likelihood of a successful live birth. There were no statistically significant associations between live birth outcomes and age (OR: 0.94; 95% CI, 0.86−1.03, *p* = 0.202) or BMI (OR: 0.95; 95% CI, 0.87−1.04, *p* = 0.260) among female participants. Male age (OR: 0.95; 95% CI, 0.87−1.04, *p* = 0.260) or male BMI (OR: 0.96; 95% CI, 0.90−1.03, *p* = 0.242) also had no statistically significant relationships with live birth outcomes. In terms of semen parameters, sperm concentration (OR: 1.04; 95% CI, 0.94−1.15, *p* = 0.435) showed no statistically significant relationship with live birth outcomes. However, TMS (OR: 1.46; 95% CI, 1.14−1.87, *p* = 0.003) had a statistically significant positive correlation with the likelihood of a successful live birth. Furthermore, although sperm morphology (OR: 1.40; 95% CI, 0.99−1.98, *p* = 0.051) did not achieve statistical significance, a trend indicating a potential effect on live birth outcomes was observed. The DFI (OR: 1.04; 95% CI, 1.01−1.08, *p* = 0.009) exhibited a statistically significant inverse relationship with the odds of achieving a live birth. A higher DFI in ejaculation was associated with an increased likelihood of a successful live birth (Table 2).

**TABLE 2:** Multiple regression analysis model for live birth.

	Factor	OR	95% CI	*p*‐value
Model	Constant			0.380
R Square 0.236	Female age	0.94	0.86−1.03	0.202
	Female BMI	0.95	0.87−1.04	0.260
	Male age	0.95	0.86−1.04	0.260
	Male BMI	0.96	0.90−1.03	0.242
	Sperm concentration	1.04	0.94−1.15	0.435
	TMS	1.46	1.14−1.87	0.003
	Sperm morphology	1.40	0.99−1.98	0.051
	DFI	1.04	1.01−1.08	0.009

Abbreviations: OR, Odds ratio; CI, confidence intervals; BMI, body mass index; TMS, total motile sperm; DFI, DNA fragmentation index.

**FIGURE 1:**
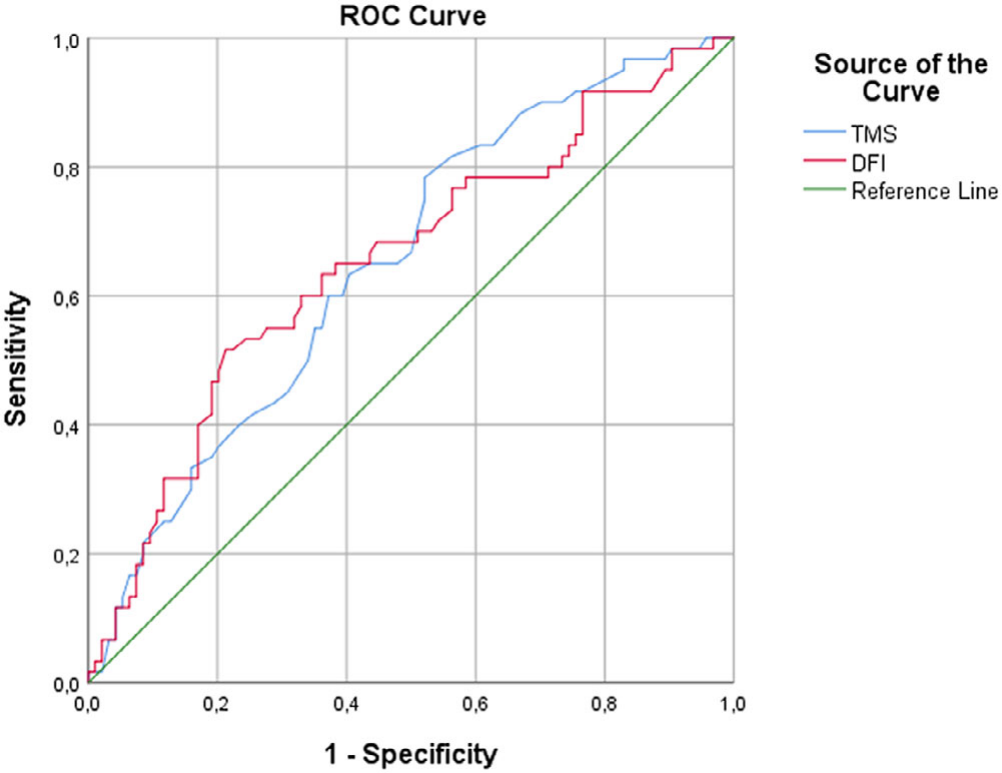
ROC curve analysis for sperm DNA fragmentation index (DFI) and total motile sperm count (TMS). ROC, Receiver operating characteristic.

The optimal sensitivity and specificity values were calculated based on the ROC curve derived from the TMS and DFI parameters. At a cutoff point of 2.55, the maximum sensitivity achieved was 78%, with a comparable specificity of 48%, yielding an AUC of 0.65. On the contrary, DFI had a maximum sensitivity of 51.7% and a specificity of 78.7% at a cutoff point of 25.8, yielding an AUC of 0.65 (Figure 1). The patients were divided into four groups based on the TMS and DFI cutoff values obtained in the ROC analysis. The probabilities of live birth in the DFI < 25.8 and TMS < 2.55, DFI < 25.8 and TMS ≥ 2.55, DFI ≥ 25.8 and TMS < 2.55, and DFI ≥ 25.8 and TMS ≥ 2.55 groups were 14.6%, 37.1%, 41.1%, and 70.6%, respectively.

Only one patient had a scrotal hematoma that did not require surgery, and no other significant complications were observed.

## 4 DISCUSSION

The findings of the present study provide valuable insights into identifying the appropriate patients who would benefit from using testicular sperm in cases of oligozoospermia, mainly when previous Ej‐ICSI cycles have been unsuccessful. We compared the characteristics of couples who had undergone T‐ICSI for the third ICSI cycle, considering whether or not live births occurred. TMS count and DFI data were significant predictors of a successful live birth outcome in the study. Our findings suggest that these parameters may help predict the likelihood of a successful outcome in cases with oligozoospermia.

The outcomes of comparative studies assessing the success of T‐ICSI and Ej‐ICSI cycles indicate significant differences. For example, Alharbi et al.^14^ conducted a retrospective review of couples with high sperm DNA fragmentation levels who underwent the T‐ICSI cycle. While the T‐ICSI group had a higher mean number of transplanted embryos, there was no statistically significant difference in clinical pregnancy and live birth rates between T‐ICSI and control groups. Nevertheless, patients in the T‐ICSI group had lower sperm concentration and motility than the other two groups. This difference in sperm quality might have impacted the results of the T‐ICSI group. Thus, while interpreting and evaluating the study's outcomes of the T‐ICSI group, the observed differences in sperm parameters should be considered.

In another study that compared two groups of patients with similar characteristics, Rauchfuss et al.^11^ found that using testicular sperm did not result in improved ICSI outcomes compared with ejaculated sperm in men with nonazoospermic male factor infertility. Specific conditions, such as recurrent IVF failure or abnormal DFI, were not included as subgroups in this study. There was no difference between the two groups because of the patients’ low DFI values; however, it is important to note that this presumption is only an estimate, as DFI was not evaluated as part of the study protocol.

In contrast to the previous studies, Esteves et al.^7^ and Zhang et al.^15^ compared the clinical outcomes of using testicular spermatozoa with ejaculated spermatozoa in ICSI‐treated infertile males with high sperm DFI. Testicular spermatozoa had higher pregnancy and delivery rates than ejaculated sperm in these studies. Herrero et al.^16^ found that T‐ICSI cycles yielded significantly higher clinical pregnancy rates and cumulative live birth rates than Ej‐ICSI cycles in couples with no previous live birth and recurrent ICSI treatment failure, particularly in cases with high sperm DNA fragmentation or abnormal semen parameters. In a retrospective cohort study, Jiang et al.^17^ compared the outcomes of using testicular sperm with ejaculated sperm in couples who had previously failed assisted reproductive techniques (ARTs) due to high rates of fragmented embryos. DFI in testicular sperm was significantly lower than in ejaculated sperm. The testicular sperm group showed significantly higher transferable embryo rate and implantation rate and lower fragmented embryo rate than the ejaculated sperm group, indicating that testicular sperm may be a better alternative for such couples.

A recent study^18^ that supports our hypothesis found a progressive and significant increase in the rate of sperm DNA damage from the testicle to the epididymis, vas deferens, and ejaculate. When comparing the DNA damage levels of ejaculated and testicular sperm from the same individuals, testicular specimens consistently had higher DNA quality.^7^, ^19^ In this context, by using testicular sperm, clinicians can avoid the limitations of ejaculated sperm and have access to a potentially healthier pool of spermatozoa, which may contribute to the embryo development and successful implantation.

In their retrospective study, Hervas et al.^20^ compared the results of Ej‐ICSI and T‐ICSI cycles within the same couple. The T‐ICSI group showed a significantly higher rate of good‐quality blastocysts than the Ej‐ICSI group. Indeed, a notable limitation in this study is the lack of reported DFI values, which indicated the positive impact of T‐ICSI cycles in patients who had previously failed ICSI attempts. Unfortunately, without this crucial information, determining whether the reported benefits of T‐ICSI cycles were related to lower testicular DFI levels in the patients becomes challenging.

Testicular sperm ICSI cycles were also better in studies involving males without oligozoospermia but had increased DFI values. In normozoospermic individuals with high sperm DFI and previous ART failure, Pabuccu et al.^21^ showed significantly better clinical and ongoing pregnancy rates and lower miscarriage rates of the T‐ICSI group than the Ej‐ICSI group. This showed that T‐ICSI cycles, even with normal sperm parameters, are an effective option for men with high sperm DFI and repeated ART failures.

A notable trend emerges upon examining the studies demonstrating the success of T‐ICSI cycles, particularly in cases involving patients with increased DFI levels. According to our findings, men who succeed with T‐ICSI cycles have high DFI levels. This finding appears to be a highly interesting and deserving area of exploration. Based on our study outcomes, T‐ICSI cycles could be routinely performed in couples with idiopathic male infertility caused by high sperm DFI values.

The nonsignificant effect of sperm morphology on fertility outcomes in our study is consistent with the literature.^22^ The main explanation for the lack of correlation between sperm morphology and ICSI cycle results is that microinjection during the ICSI cycle allows some of the obstacles faced during natural processes to be overcome. Additionally, the sperm used in ICSI cycles does not fully represent the total population.

TMS may be more predictive of live birth outcomes in patients undergoing T‐ICSI cycles than sperm concentration. Bole et al.^23^ conducted a study on 23 couples who had T‐ICSI cycles, which was similar to our study. Couples with male partners having TMS > 10 million had an 85.7% pregnancy rate compared with 25% for TMS ≤10 million (*p* = 0.007). TMS > 10 million predicted biochemical pregnancy. Increased focus on TMS rather than sperm concentration has the potential to yield more valuable and appreciated data.

Esteves et al.^7^ reported a 6.2% complication rate after testicular sperm extraction (TESE) or TESA sperm retrieval, with pain being the most common complication noted. Similarly, our study found a comparable and satisfactory complication rate with TESA sperm retrieval. While the high live birth rate is promising, it is crucial to recognize that testicular sperm retrieval has specific risks, including potential testicular tissue damage and complications, such as bleeding or infection. Therefore, careful patient selection, adherence to best practices during the retrieval procedure, and performance of the procedure by skilled and experienced professionals are essential to minimize potential risks.

Because sperm retrieval is a more intrusive and costly method in the T‐ICSI cycle than in the Ej‐ICSI cycle, predicting the patient group with high T‐ICSI cycle success may make it simpler for us to decide on T‐ICSI treatment. Despite the limitation provided by a relatively small‐sample size in our study group, an observed live birth rate of 70.6% was found in patients with TMS and DFI values beyond the corresponding cutoff limits for both parameters. This finding shows that T‐ICSI treatment may be recommended in such cases. Conversely, the option to proceed with Ej‐ICSI cycles may be considered for patients with lower TMS and DFI values. The notably high success rate might be related to the small number of participants in the study.

Our study has several limitations. First, a retrospective study at a single center limits the sample size. Second, due to the retrospective nature of the study, the DFI value was measured in the ejaculate, and it was not assessed in the testicular tissue. Third, although we excluded cases of female factor‐induced infertility, unexplained female infertility cannot be ruled out. Fourth, there was no control group in our study. However, it is essential to note that the primary objective of our study was to predict the success of T‐ICSI cycles in couples who had failed Ej‐ICSI cycles; thus, a control group was unnecessary. Lastly, the fact that the study includes men with oligozoospermia and idiopathic male infertility might create the impression that we are focusing on a relatively limited group of patients, and our findings may not be generalizable to all cases of male infertility.

## 5 CONCLUSION

In conclusion, for individuals who have had recurrent failures with Ej‐ICSI procedures, higher DFI and TMS in the ejaculate may be helpful to predictive indicators for the success of subsequent T‐ICSI attempts. When high DFI and TMS are observed in patients with a history of unsuccessful Ej‐ICSI cycles, testicular sperm can be used for future ICSI procedures. DFI measurement in ejaculation has a significant advantage over testicular DFI assessment as it is noninvasive. This paradigm change toward T‐ICSI cycles, guided by these indicators, presents a viable option for improving the chances of successful fertility treatment in cases when Ej‐ICSI cycles have proven ineffective.

## AUTHOR CONTRIBUTIONS

Muhammed Arif Ibis and Eda Ureyen Ozdemir performed the research, Cagri Akpinar and Khaled Obaid analyzed the data and wrote the paper, and Batuhan Ozmen, Khaled Obaid, and Onder Yaman designed the research study and wrote the paper.

## CONFLICT OF INTEREST STATEMENT

The authors declare no conflicts of interest.

## Data Availability

Data available on request from the authors.

## References

[1] Salonia ABC , Capogrosso P , Carvalho J , et al. EAU Guidelines on Sexual and Reproductive Health. 2023. https://d56bochluxqnz.cloudfront.net/documents/full‐guideline/EAU‐Guidelines‐on‐Sexual‐and‐Reproductive‐Health‐2023.pdf

[2] Lopes LS , Esteves SC . Testicular sperm for intracytoplasmic sperm injection in non‐azoospermic men: a paradigm shift. Panminerva Med. 2019;61(2):178‐186. 30990286 10.23736/S0031-0808.18.03534-6

[3] Esteves SC , Coimbra I , Hallak J . Surgically retrieved spermatozoa for ICSI cycles in non‐azoospermic males with high sperm DNA fragmentation in semen. Andrologia. 2023;11(8):1613‐1634. 10.1111/andr.13405 36734283

[4] Evgeni E , Sabbaghian M , Saleh R , et al. Sperm DNA fragmentation test: usefulness in assessing male fertility and assisted reproductive technology outcomes. Panminerva Med. 2023;65(2):135‐147. 37103485 10.23736/S0031-0808.23.04836-X

[5] Santi D , Spaggiari G , Simoni M . Sperm DNA fragmentation index as a promising predictive tool for male infertility diagnosis and treatment management–meta‐analyses. Reprod Biomed Online. 2018;37(3):315‐326. 30314886 10.1016/j.rbmo.2018.06.023

[6] Okubo T , Onda N , Hayashi T , Kobayashi T , Omi K , Segawa T . Performing a sperm DNA fragmentation test in addition to semen examination based on the WHO criteria can be a more accurate diagnosis of IVF outcomes. BMC Urol. 2023;23(1):78. 37120514 10.1186/s12894-023-01257-y PMC10148994

[7] Esteves SC , Sánchez‐Martín F , Sánchez‐Martín P , Schneider DT , Gosálvez J . Comparison of reproductive outcome in oligozoospermic men with high sperm DNA fragmentation undergoing intracytoplasmic sperm injection with ejaculated and testicular sperm. Fertil Steril. 2015;104(6):1398‐1405. 26428305 10.1016/j.fertnstert.2015.08.028

[8] Ariel M , Cedar H , McCarrey J . Developmental changes in methylation of spermatogenesis‐specific genes include reprogramming in the epididymis. Nat Genet. 1994;7(1):59‐63. 8075642 10.1038/ng0594-59

[9] O'Flaherty C , Scarlata E . Oxidative stress and reproductive function: the protection of mammalian spermatozoa against oxidative stress. Reproduction. 2022;164(6):F67‐F78. 37021966 10.1530/REP-22-0200

[10] Waheed A , Rai MF . Year in review 2023: genetics, genomics, and epigenetics. Osteoarthr Cartil. 2023;32(2):128‐137. 10.1016/j.joca.2023.11.006 37979669

[11] Rauchfuss LMK , Kim T , Bleess JL , Ziegelmann MJ , Shenoy CC . Testicular sperm extraction vs. ejaculated sperm use for nonazoospermic male factor infertility. Fertil Steril. 2021;116(4):963‐970. 34233843 10.1016/j.fertnstert.2021.05.087

[12] Kang Y‐N , Hsiao Y‐W , Chen C‐Y , Wu C‐C . Testicular sperm is superior to ejaculated sperm for ICSI in cryptozoospermia: an update systematic review and meta‐analysis. Sci Rep. 2018;8(1):7874. 29777145 10.1038/s41598-018-26280-0 PMC5959851

[13] Turek PJ , Cha I , Ljung B‐M . Systematic fine‐needle aspiration of the testis: correlation to biopsy and results of organ “mapping” for mature sperm in azoospermic men. Urology. 1997;49(5):743‐748. 9145981 10.1016/S0090-4295(97)00154-4

[14] Alharbi M , Hamouche F , Phillips S , Kadoch JI , Zini A . Use of testicular sperm in couples with SCSA‐defined high sperm DNA fragmentation and failed intracytoplasmic sperm injection using ejaculated sperm. Asian J Androl. 2020;22(4):348. 31571640 10.4103/aja.aja_99_19 PMC7406103

[15] Zhang J , Xue H , Qiu F , Zhong J , Su J . Testicular spermatozoon is superior to ejaculated spermatozoon for intracytoplasmic sperm injection to achieve pregnancy in infertile males with high sperm DNA damage. Andrologia. 2019;51(2):e13175. 30474187 10.1111/and.13175

[16] Herrero M , Lusignan M , Son WY , Sabbah M , Buckett W , Chan P . ICSI outcomes using testicular spermatozoa in non‐azoospermic couples with recurrent ICSI failure and no previous live births. Andrologia. 2019;7(3):281‐287. 10.1111/andr.12591 30734539

[17] Jiang L‐Y , Kong F‐F , Yao L , et al. Are testicular sperms superior to ejaculated sperms in couples with previous ART failure due to high rate of fragmented embryos? A retrospective cohort study. Front Surgery. 2023;9:1065751. 10.3389/fsurg.2022.1065751 PMC9852334 36684174

[18] Xie P , Keating D , Parrella A , et al. Sperm genomic integrity by TUNEL varies throughout the male genital tract. J Urol. 2020;203(4):802‐808. 31738116 10.1097/JU.0000000000000659

[19] Mehta A , Bolyakov A , Schlegel PN , Paduch DA . Higher pregnancy rates using testicular sperm in men with severe oligospermia. Fertil Steril. 2015;104(6):1382‐1387. 26363389 10.1016/j.fertnstert.2015.08.008

[20] Hervas I , Gil Julia M , Rivera‐Egea R , Navarro‐Gomezlechon A , Mossetti L , Garrido N . Switching to testicular sperm after a previous ICSI failure with ejaculated sperm significantly improves blastocyst quality without increasing aneuploidy risk. J Assist Reprod Genet. 2022;39(10):2275‐2285. 35972585 10.1007/s10815-022-02595-w PMC9596654

[21] Pabuçcu EG , Caglar G , Tangal S , Haliloglu A , Pabuçcu R . Testicular versus ejaculated spermatozoa in ICSI cycles of normozoospermic men with high sperm DNA fragmentation and previous ART failures. Andrologia. 2017;49(2):e12609. 10.1111/and.12609 27108915

[22] Gatimel N , Moreau J , Parinaud J , Léandri R . Sperm morphology: assessment, pathophysiology, clinical relevance, and state of the art in 2017. Andrologia. 2017;5(5):845‐862. 10.1111/andr.12389 28692759

[23] Bole R , Yang D , Ziegelmann M , et al. Total motile sperm count is associated with ICSI success using sperm obtained by TESE. Fertil Steril. 2021;116(3):e24.

